# Configurational entropy of ice XIX and its isotope effect

**DOI:** 10.1038/s41598-024-61250-9

**Published:** 2024-05-07

**Authors:** Tobias M. Gasser, Alexander V. Thoeny, A. Dominic Fortes, Thomas Loerting

**Affiliations:** 1 Institute of Physical Chemistry, University of Innsbruck, 6020 Innsbruck, Austria; 2 ISIS Neutron and Muon Facility, Rutherford Appleton Laboratory, Harwell Science and Innovation Campus, Chilton, Oxfordshire OX11 0QX UK

**Keywords:** Chemical physics, Reaction kinetics and dynamics, Thermodynamics, Phase transitions and critical phenomena, Chemical physics, Cryospheric science

## Abstract

Ice XIX is a partly hydrogen-ordered polymorph related to disordered ice VI, similar to ice XV. We here investigate the order–order–disorder sequence ice XIX→ice XV→ice VI based on calorimetry at ambient pressure both for D_2_O and H_2_O-ice XIX. From these data we extract configurational entropy differences between ice XIX, ice XV and ice VI. This task is complex because, unlike for all other ices, the order–disorder transition from ice XIX to ice VI takes place in two steps via ice XV. Even more challenging, these two steps take place in an overlapping manner, so that careful separation of slow kinetics is necessary. This is evidenced best by changing the heating rate in calorimetry experiments: For fast heating experiments the second step, disordering of ice XV, is suppressed because the first step, formation of ice XV from ice XIX, is too slow. The transient state ice VI^‡^ that is initially produced upon ice XIX decay then does not have enough time to convert to ice XV, but remains disordered all along. In order to tackle the challenge to determine the entropy difference between ice XIX and VI as well as the entropy difference between ice XV and VI we employ two different approaches that allow assessing the impact of kinetics on the entropy change. “Single peak integration” defines a kinetically limited result, but “combined peak integration” allows estimation of the true thermodynamic values. Our best estimate for the true value shows ice XIX to be much more ordered than ice XV (25 ± 3% vs 9 ± 4% of the Pauling entropy). For D_2_Oice XIX samples we obtain 28% of order, but only when a small number of fast H-isotope defects are used. In the second part we use these results to estimate the location of the ice XIX phase boundary both for protiated and deuterated ice XIX. The initial Clapeyron slope at ambient pressure is determined from the combination of neutron powder diffraction volume differences and calorimetry entropy differences data to be 21 K GPa^−1^ with an order–disorder transition temperature *T*_*o-d*_(0.0 GPa) = 103 ± 1 K. An in situ bracketing experiment at 1.8 GPa yields *T*_*o-d*_(1.8 GPa) = 116 ± 3 K, i.e., the phase boundary slope flattens at higher pressures. These data allow us to determine the region of thermodynamic stability of ice XIX in the phase diagram and to explain the surprising isotope shift reversal at 1.6 GPa compared to 0.0 GPa, i.e., why D_2_O-ice XIX disorders at lower temperatures than H_2_O-ice XIX at 1.6 GPa, but at higher temperatures at ambient pressures.

## Introduction

The astonishingly large number of different ice polymorphs is one of water’s many anomalies. Different ice polymorphs differ from each other in terms of space group and unit cell^1^. Some of the ice polymorphs differ significantly in terms of mass density. In such cases the lattice of oxygen atoms is different, different types of rings are encountered, and for some ices such as ice VI two interpenetrating H-bond networks (that are not connected with each other) cause the higher density. Other ice polymorphs show very similar densities, deviating less than 1% from each other^1^. One example for this situation is hexagonal (ice I_h_) and cubic ice (ice I_c_). In this case both ices are composed of layers of six-membered rings of O-atoms, where only the sequence of layers differs. This is known as polytypism, a sub-category of polymorphism^2–4^. In all other examples for ices of very similar density the O-atom topology of both ices is identical, but the arrangement of H-atoms is distinct. The sublattice of H-atoms maybe fully ordered, fully disordered or partially ordered within a given lattice of O-atoms. This is equivalent to ordered or disordered dipolar orientations of water molecules in the lattice, where the ordering may give rise to anti-ferroelectric properties and prevents the flow or creep of ice that is well-known for (disordered) ice I_h_ in glaciers^5,6^. Such ices usually appear in pairs of one ordered and one disordered polymorph, which differ in terms of configurational entropy of water molecules, but barely in density. Based on the third law of thermodynamics the stable ice at (ultra)low temperatures should be fully ordered. In practice, ordered ices are typically not fully, but only partially ordered (such as ice XI or ice XV)—and the thermodynamically most stable one is the one with the highest degree of order, where highest order is equivalent to lowest configurational entropy. Phase boundaries between such pairs of ices are parallel to the pressure axis because of the (near) zero difference in volume between the two ices. For example this is the case for the ice I_h_/XI transition or ice III/IX. By contrast, phase boundaries are almost parallel to the temperature axis if the O-atom topology changes at the transition between two disordered ices. The ice III/V phase boundary and the ice V/VI phase boundary are examples for this kind of situation. These phase boundaries are of great importance in understanding icy bodies in the universe, e.g., the icy mantles of Saturn or Jupiter, which are often covered in layers of ices I, II, III/IX, V/XIII and VI/XV/XIX. Just like for glaciers on Earth it is important to understand the flow properties and brittleness of theses layers. This in turn is dominated by the question whether or not the water molecules are ordered—a question that we here address on the example of ices VI/XV/XIX.

Ice XIX is the latest ice polymorph to be discovered. It represents a partially ordered counterpart to disordered ice VI. Also ice XV represents a partially ordered counterpart to disordered ice VI, which makes ice XIX/XV/VI the first trio sharing the same network of oxygen atoms. We claim ice XIX to show a stability domain at low temperature and high pressure in the phase diagram of water because of its higher degree of order. Yet, the configurational entropy of these ices has not been quantitatively determined up to now, and so the location of the phase boundary in the phase diagram is still unclear^7,8^. For this reason we here tackle this issue and attempt to determine the volume differences Δ*V* and the configurational entropy differences Δ*S* within this trio. This then allows us to calculate the slope of the phase boundary as a Clapeyron slope d*T*/d*p* = Δ*V*/Δ*S*. The first evidence for the existence of ice XIX in experiments on pressurized H_2_O samples was presented in 2018 by some of us^9^, where we show that this ice phase shares the same network of oxygen atoms with ice VI and ice XV, but differs in terms of its statistical arrangement of hydrogen atoms. This evidence is based on X-ray powder diffraction, calorimetry, Raman and dielectric relaxation spectroscopy. Neutron powder diffraction experiments were then required in order to characterise accurately the structure of ice XIX. This method ideally requires perdeuterated material to determine the positions of the light atoms in the unit cell. A key issue that had to be resolved first was the effect of deuteration on ice XIX formation. For most ice polymorphs the strategy to make the deuterated polymorph is quite simple: replacing H_2_O by D_2_O and following the same experimental protocols. In the case of ice XIX it turns out that this strategy does not work. While protiated ice XIX does form upon cooling ice VI at 3 K min^−1^ at 1.8 GPa, deuterated ice XIX does not. As an explanation for this unexpected result Gasser et al.^9^ hypothesized that the mobility of the deuterium atoms is too low to adopt the positions in ice XIX. Attempts to simply cool more slowly and provide more time for the deuterium atoms to reach their positions in ice XIX failed. Even six times slower cooling rates of 0.5 K min^−1^ or 25 h of annealing at 105 K do not provide enough time for transformation to occur in deuterated material^9^. We subsequently discovered that the successful strategy to produce deuterated ice XIX is to add a small amount of H_2_O to D_2_O: just 0.5% H_2_O is sufficient to produce deuterated ice XIX at cooling rates of 3 K min^−1^^7^. In other words, the addition of a small amount of more mobile H-atoms in the bath of D-atoms massively accelerates the reorientation dynamics in the D-atom sublattice. This strategy ultimately has made 99.5% deuterated samples available for neutron powder diffraction experiments and paved the way for the determination of the ice XIX crystal structure. The best fit to the neutron data reveals a $P\overline{4 }$ structure model involving a partly antiferroelectrically ordered lattice of D_2_O molecules^7^. Yamane et al.^8^ independently reached the same conclusion and found the same structural model. While the ice XIX structure determined by Gasser et al. was determined for an ex situ sample at ambient pressure (after recovery from the high-pressure cell), the ice XIX structure determined by Yamane et al. was determined for an in situ sample at 1.6 GPa inside the pressure cell.

The unit cell of ice XIX contains twenty D-atoms and ten distinct hydrogen-bonds connecting adjacent oxygen atoms. The Bernal-Fowler ice rules^10^ require that the sum of the occupancies of two D atoms in one hydrogen-bond is exactly 1.0. For a fully disordered ice all occupancies are 0.50/0.50, and for a fully ordered ice all occupancies are 1.0/0.0. In ice XIX three hydrogen bonds are fully disordered within the unit cell, whereas the other seven hydrogen bonds are partly ordered. In the 1.6 GPa ice XIX structure refined by Yamane et al. these seven hydrogen bonds show average occupancies of 0.672/0.328^8^. Similarly, the refined structure of recovered ice XIX (at subambient pressure) shows average occupancies of 0.711/0.289^7^. When defining the difference between the two occupancies as the “diffraction-inferred order” (DIO), the neutron result is 42% DIO at subambient pressure and 34% DIO at 1.6 GPa. Soon after the refinement of the ice XIX structure by Yamane et al. and Gasser et al. a third neutron diffraction study on the “ice XIX” structure was presented by Salzmann et al.^11^. Although these authors find the same supercell as Yamane et al. and Gasser et al., they adopted a structural model in space group *Pbcn* on the basis of a Reverse Monte Carlo analysis of their data. There are two important differences between their model and the two previously published structures. First, and most importantly they refine their data based on a fully orientationally disordered model. All occupancies are 0.50/0.50, and their DIO is 0% for an ice sample studied in situ at 1.6 GPa. Secondly, their model explains the development of a 2 × 2 × 1 supercell by the collective tilting and distortion of the hexameric O-atom units. Consequently, their “ice XIX” is not favoured in terms of configurational entropy over ice XV, so that “ice XIX” would not be a thermodynamically stable low-temperature and high-pressure phase of ice. We argue that Salzmann et al. in fact did not produce ice XIX in their neutron study, but a distorted form of ice VI, because they were missing the key ingredient that allows the D-atoms to order. They did not add a tiny amount of hydrogen to their deuterated sample, so that the mobility in the D-sublattice was simply too low to actually reach a DIO different from 0%. That is, we claim that the isotopic composition has a massive effect on the degree of order that develops upon cooling ice VI. Similar conclusions that the “ice XIX” claimed by Salzmann et al. is not really ice XIX were reached by Komatsu et al. in a comprehensive review on the topic^12^. In the present work we investigate the degree of order for both deuterated and protiated samples based on differential scanning calorimetry experiments on both ice XV and ice XIX in detail. We introduce the concept of “calorimetrically-inferred order” (CIO) that is directly linked to the configurational entropy *S*_conf_. According to Pauling^13^ a fully disordered, ringless ice lattice is defined through *S*_conf_ = R*ln(3/2) = 3.371 J mol^−1^ K^−1^^13^. This approximation has proven to be quite accurate also for ice polymorphs containing rings, such as hexagonal ice (ice I_h_) or ice VI^14^. A fully ordered ice, by contrast, shows *S*_conf_ = 0, in accordance with the third law of thermodynamics. In other words, we here examine the question whether or not ice XIX is favoured over ice XV in terms of *S*_conf_ and how *S*_conf_ changes upon full or partial deuteration of the H_2_O sample. This then allows us to compare CIO and DIO for differently prepared variants of ice XIX. Please note in this context that there is no linear relation between the order defined based on the occupancies derived from diffraction experiments and the order derived from heat evolution observations in calorimetry experiments. In fact there is no known quantitiative relation between the two, so that we here merely qualitatively compare the two.

We estimate the Clapeyron slope of the order–disorder transition temperature *T*_*o-d*_(XIX/VI) based on the volume difference between ice VI and ice XIX as deduced from our previous neutron diffraction experiment^1^ and based on the entropy difference based on the calorimetry experiment reported here, both done at (sub)ambient pressure. This allows us to define the initial slope of the ice XIX/VI phase boundary at 0.0 GPa. A comparison of Clapeyron slope between protiated and deuterated samples furthermore allows us to explain the reverted isotope effect under high pressure conditions, i.e., why Yamane et al.^8^ find *T*_*o-d*_(H_2_O ice XIX) > *T*_*o-d*_(D_2_O ice XIX) from dielectric experiments at 1.6 GPa, whereas it is the other way around at ambient pressure in our calorimetry experiments. Finally, we investigate the pressure-dependence of the calorimetric order–disorder temperature *T*_*o-d*_ for ice XIX between ambient pressure and 1.8 GPa. To this end we carry out an in situ experiment aimed at bracketing *T*_*o-d*_ of ice XIX at 1.8 GPa. This allows us to ultimately intrapolate the location of the phase boundary and to suggest the stability domain of ice XIX in the phase diagram. We note, though, that the definition of the phase boundary associated with ice XIX disordering requires thermodynamic equilibrium, which is difficult to attain in experiments as alluded to below in the results section. That is, the result in the phase diagram represents the best estimate we can do, but still requires more work—especially more in situ experiments in the range between 0.5 and 1.5 GPa will be very useful to define the phase boundary fully on the basis of experiments rather than on intrapolation of experiments done at 0.0 GPa and 1.8 GPa only.

## Experimental

Ice XIX and ice XV sample preparation was done in the same way as described in our earlier work^7,9,15,16^. In brief, 600 µl of H_2_O (or D_2_O or H_2_O/D_2_O mixtures) containing 0.01 mol per liter HCl (or DCl) as dopant were lined with an indium foil and inserted into the 8 mm bore of a steel cylinder that is kept at 77 K. This leads to freezing of hexagonal ice, which is first compressed to 1.8 GPa using a piston-cylinder setup in our universal testing machine ZWICK BZ100/TL3S and then heated to 255 K. This causes amorphization of the ice, followed by the crystallization of the metastable high-pressure polymorph ice XII and finally the polymorphic transformation to stable ice VI. Ice VI is then cooled between 160 and 90 K at ≤ 3 K min^−1^ at 1.8 GPa, which leads to the formation of ice XIX. In this work we have used pure H_2_O, pure D_2_O (> 99.96%), and two different isotope mixtures: 95% H_2_O/5% D_2_O (labelled 95% H) and 5% H_2_O/95% D_2_O (labelled 5% H).

These samples are then quenched to 77 K, recovered from the steel cylinder, stored in liquid nitrogen and transferred to the calorimeter, X-ray diffractometer or neutron diffractometer for sample characterization. The samples are manipulated while immersed in liquid nitrogen, and the transfer always takes place to sample holders, which are kept below 80 K.

For calorimetry and diffraction experiments, small chunks were cut from the sample cylinder. Calorimetric characterization was done using a Perkin Elmer DSC 8000 differential scanning calorimeter, where about 10–20 mg of ice were filled into aluminium crucibles under liquid nitrogen and transferred to the precooled oven of the calorimeter. Each sample was heated to 253 K at 10 K min^−1^. The resulting ice I_h_ was cooled to 93 K and heated a second time to 313 K. The second, featureless (up to 273 K) heating run was employed as baseline for the first scan. To determine the exact sample mass, the area under the ice melting endotherm (above 273 K) was used. For X-ray characterization 30–100 mg were powdered and placed on the sample holder at 80 K and recorded with a Bruker D8 Advance powder diffractometer with a Cu sample holder equipped with a PheniX Helium Cryostat (measurements at 20 K), a Cu-Kα X-ray source and using a Göbel-mirror for parallel beam or Bragg–Brentano optics. The unit cell volume of ice VI and ice XIX were obtained on the HRPD instrument at the Rutherford Appleton Laboratory using the techniques described in our earlier work^7^. X-ray diffractograms demonstrating that all the samples are ice XV/XIX are shown in Figs. S3–S5 in the Supporting Information.

Calorimetry scans are analyzed in terms of enthalpies of transformation Δ*H* from the peak area and onset temperatures for phase transition *T*_*o-d*_, which are known to be independent of the scan rate, i.e., not to be affected by transition kinetics. The configurational entropy is then calculated from these two by simple division. Please note that this calculation is under the assumption that *T*_*o-d*_ represents the equilibrium transition temperature for the order–disorder transition. Even though ice XIX disordering can only be reversed above 1 GPa, but not at ambient pressure, we regard *T*_*o-d*_(XIX), as well as *T*_*o-d*_(XV), to be a reasonable estimate for the equilibrium temperature as detailed below. For other order-disorder transitions in ice phases such as for ice XIV/XII and ice XIII/V, it was shown that the onset temperature of disordering upon heating matches very well with the onset temperature of ordering upon cooling, i.e., the process is in equilibrium in spite of the kinetic limitations at temperatures near 100 K.

## Results

### Heating rate dependence of H-ordering and H-disordering transitions in ice XIX

**Figure 1:**
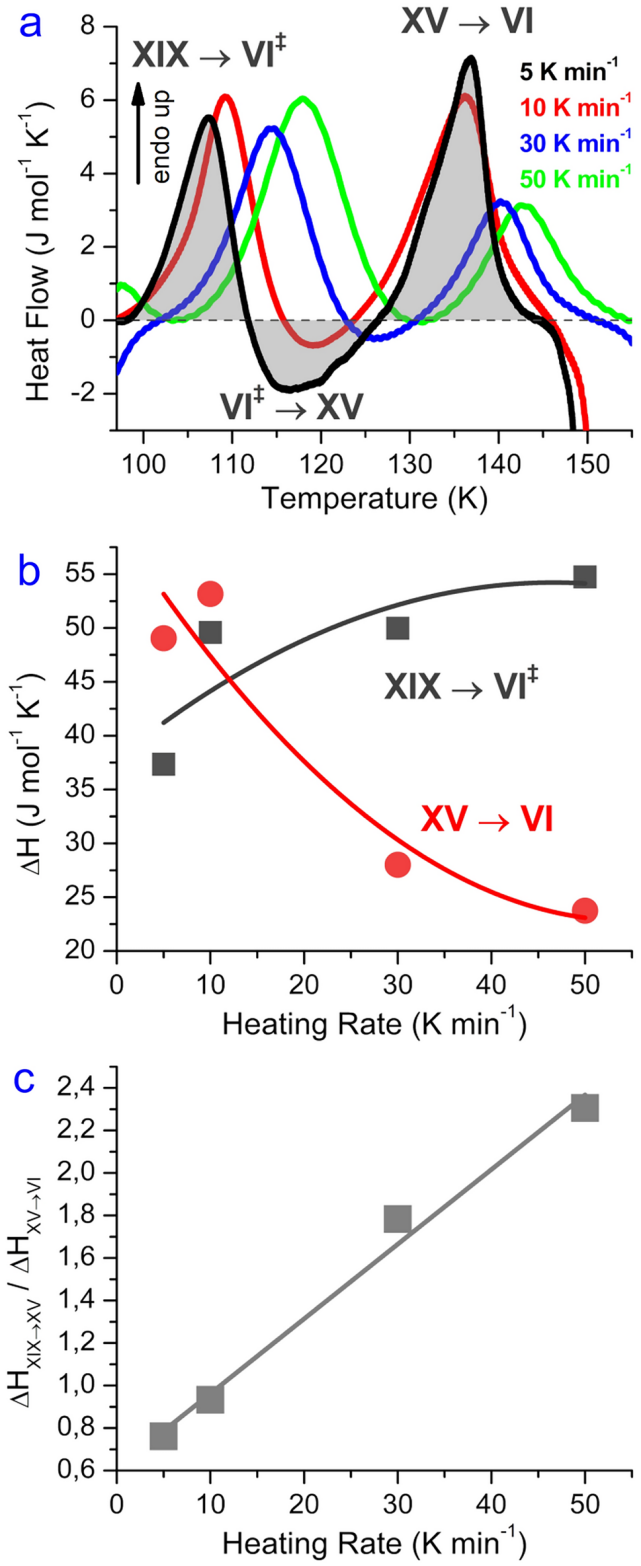
Kinetic effect of heating rates on enthalpies of transition sequence Ice XIX → Ice VI^‡^ → Ice XV → Ice VI. (**a**) Calorigrams recorded at 5 (black trace), 10 (red trace), 30 (blue trace) and 50 (green trace) K min^−1^ scan rate. The grey dashed line represents the baseline, the grey shaded areas illustrate the calculation of the peak enthalpies. (**b**) Peak enthalpies and (**c**) peak ratios (grey dots) of disordering transitions XIX → VI^‡^ (black dots) and XV → VI (red dots) at different heating rates. Lines in (**b**) and (**c**) are guides to the eyes. Pure H_2_O ice XIX samples prepared at 1.8 GPa were used for the experiments. Endothermic peaks are pointing upwards.

Figure 1 shows calorimetry traces obtained at different heating rates from 5 to 50 K min^−1^. It is immediately evident that the traces are quite different. All scans show two endothermic features (above the grey dashed baseline), but their position and size differ. Only some of the scans show exothermic features (below the grey dashed baseline). The three features are highlighted in grey for the trace recorded at 5 K min^−1^ in Fig. 1a. We attribute these three processes to the (transient) disordering of ice XIX (ca. 100–112 K), ordering of the transiently disordered ice to ice XV (ca. 112–126 K) and disordering of ice XV (ca. 126–142 K), producing ice VI. For all these transitions the O-atom network remains the same. At about 145 K a massive heat release is incurred, which corresponds to the rearrangement of ice VI to ice I, where the density changes by about 30%. Only the onset of this transition is seen in Fig. 1a.

At higher heating rates all the transition temperatures mentioned above shift to higher temperatures, as usual in calorimetry experiments. More importantly, though, also the size of the individual three peaks changes dramatically. The size of the ice VI^‡^→ice XV exotherm shrinks significantly and reaches approximately zero at 50 K min^−1^. Also the size of the ice XV→ice VI endotherm shrinks massively with heating rate. The heat taken up at the ice XV H-disordering transition is roughly halved from about 50 J mol^−1^ to about 25 J mol^−1^ as shown in Fig. 1b. At the same time the first exotherm pertaining to ice XIX H-disordering increases from about 40 J mol^−1^ to about 55 J mol^−1^. That is, the dominant heat contribution at 5 K min^−1^ originates from ice XV disordering, but at 50 K min^−1^ the ice XIX disordering endotherm is larger by a factor of 2.4 than the ice XV disordering endotherm. This clearly indicates the kinetic effect and the fact that we are dealing with a difficult kinetic problem. We have three fundamental steps, each of which features its own kinetics. Even more importantly these processes rely on the ice to be delivered from the previous step. These kinetics are all very slow due to the low temperature and take place simultaneously. For example, ice VI^‡^ is produced from ice XIX but simultaneously is depleted by conversion to ice XV. The ice VI^‡^ that is not converted once a temperature of 140 K has been reached remains ice VI. Whereas ice VI is a transient state at 100 K, it is a stable state at 140 K due to entropy gaining more importance at higher temperature. That is, it is very hard to disentangle the kinetics aspects from the true thermodynamic aspect. Clearly, heat evolution from different processes overlaps, where exothermic and endothermic processes take place simultaneously and annihilate each other. That means the individual peaks as depicted in grey in Fig. 1a are kinetically determined, but not thermodynamically. In order to account for the thermodynamic situation this peak overlap needs to be removed, and the enthalpy of the ice XIX→ice VI transition needs to be calculated disregarding the peak overlap. The same holds true for the ice XV→ice VI transition—we need to define the transition enthalpy in the limit of infinitely slow kinetics, which then also allows us to calculate the entropy change from the order–disorder transition temperature *T*_o-d_ in this limit.

### Comparison of ice XV and ice XIX thermograms including isotope effect

**Figure 2:**
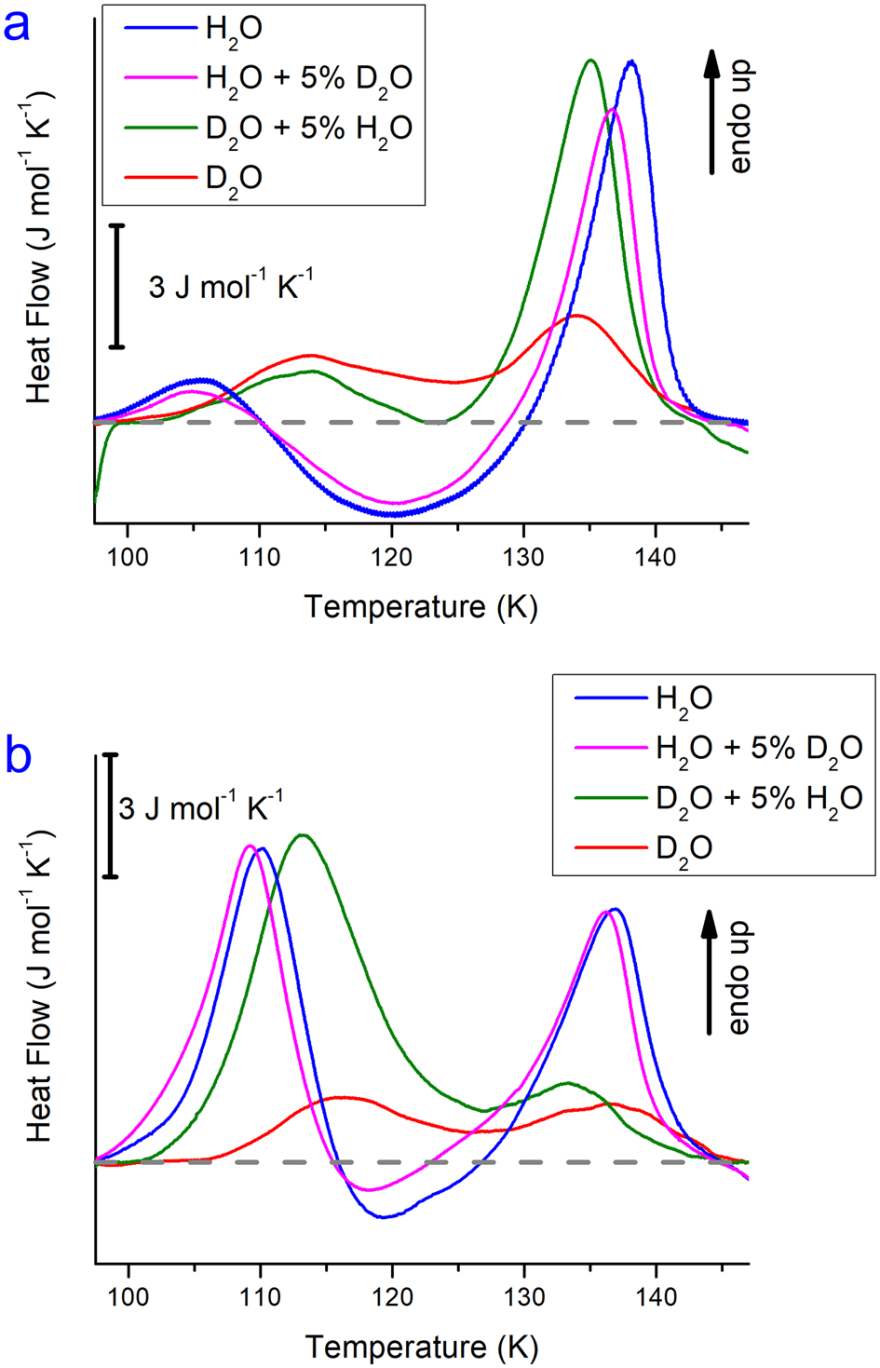
Impact of H/D isotope substitution on calorimetry scans for (**a**) ice XV prepared at 1.0 GPa and (**b**) ice XIX prepared at 1.8 GPa. All scans were recorded at 10 K min^−1^. The grey dashed line represents the baseline. D_2_O and D_2_O + 5% H_2_O samples were cooled at 0.5 K min^−1^, whereas H_2_O and H_2_O + 5% D_2_O were cooled faster (3 K min^−1^ for ice XIX, and > 40 K min^−1^ for ice XV).

Figure 2 shows calorigrams for ice XV (panel a) and ice XIX (panel b) for the different isotopic mixtures, namely pure H_2_O (blue), 5% deuterated H_2_O (purple), pure D_2_O (red) and 5% protiated D_2_O (green). Both for ice XV and for ice XIX the fully deuterated samples (red traces) show smaller endotherms than all other isotopologues, in spite of the lowest cooling rates used to make them (0.5 K min^−1^). This directly suggests that fully deuterated ices XV and XIX are less ordered than partly or fully protiated samples. Table 1 lists the peak areas for the three features in the scan: the first endotherm, Δ*H*(XIX), the exotherm, Δ*H*(VI^‡^), and the second endotherm, Δ*H*(XV). Table 1 also lists the onset temperatures for the two endotherms, which are recognized as onsets for ice XIX and ice XV disordering, *T*_*o-d*_(XIX) and *T*_*o-d*_(XV), respectively.

**Table 1:** Summary of all onset temperatures *T*_*o-d*_ and enthalpy changes Δ*H* extracted from the collected calorimetry traces in this work.

H/D	*T*_*o-d*_(XIX) (K)	*T*_*o-d*_(XV) (K)	Δ*H*(XIX) (J mol^−1^)	Δ*H*(VI^‡^) (J mol^−1^)	Δ*H*(XV) (J mol^−1^)	Δ*H*_tot_ (J mol^−1^)
*Ice XV (1.0 GPa)*						
100% H	99.9 ± 0.7 (4/12)	131.3 ± 0.8 (5/17)	4 ± 4 (5/15)	− 38 ± 11 (5/16)	58 ± 9 (5/16)	39 ± 19 (4/11)
	–	128 [Fig. 2 in Ref.^21^]	–	− 64.4 [Ref.^21^]	112.0 [Ref.^21^]	47.6 [Calculated from ref.^21^]
95% H	100.3 ± 0.9 (6/11)	131.2 ± 0.8 (8/16)	6 ± 5 (8/19)	− 24 ± 11 (8/18)	44 ± 10 (8/19)	27 ± 13 (8/16)
5% H	106 ± 3 (5/11)	130 ± 2 (5/11)	14 ± 5 (5/12)	–	36 ± 10 (5/12)	63 ± 13 (5/10)
0% H	105 ± 2 (4/7)	126 ± 4 (4/7)	4 ± 4 (4/8)	–	10 ± 7 (4/9)	26 ± 9 (3/6)
	–	126 [Fig. 2 in Ref.^21^]	–	–	24.1 [Ref.^21^]	24.1 [Ref.^21^]
*Ice XIX (1.8 GPa)*						
100% H	103.2 ± 0.8 (8/21)	128 ± 2 (8/18)	50 ± 8 (8/21)	− 6 ± 3 (8/19)	44 ± 6 (8/21)	87 ± 11 (8/21)
95% H	104 ± 1 (14/36)	129 ± 2 (13/34)	42 ± 11 (14/36)	Ambiguous	Ambiguous	72 ± 15 (13/33)
5% H	106.3 ± 0.6 (21/31)	118 ± 3 (22/31)	72 ± 5 (22/32)	Ambiguous	Ambiguous	101 ± 8 (22/33)
0% H	108 ± 2 (7/17)	124 ± 5 (7/17)	7 ± 3 (7/17)	Ambiguous	Ambiguous	17 ± 6 (7/16)

Two types of approaches were used to extract the peak areas: (i) the “single peak integration approach”, in which both endotherms and exotherms were integrated individually (columns Δ*H*(XIX), Δ*H*(VI^‡^) and Δ*H*(XV)), and (ii) the “combined integration approach” (column Δ*H*_tot_) , in which the integration is done from *T*_*o-d*_(XIX) till the offset of the XV/VI transition (near 145 K). Values from the work by Salzmann et al. were extracted from the graphs shown in ref.^21^. The error provided for each entry represents the standard deviation. The number of batches b used to determine the average and the total number of scans n is given as “(b/n)”, i.e., “(7/17)” means that seven ice XIX samples were prepared, and in total 17 DSC crucibles were filled and scanned. To calculate configurational entropy for ices XV and XIX one needs to equate *S*_conf_(XV) = Δ*H*_tot_/*T*_*o-d*_(XV) and *S*_conf_(XIX)≈ Δ*H*_tot_/*T*_*o-d*_(XIX).

Let us now look specifically at the collected data for ice XV samples prepared through cooling ice VI at 1.0 GPa (Fig. 2a). As demonstrated in our previous work^16^ such samples contain mainly ice XV domains, but also a minority of ice XIX domains. These ice XIX domains cause the first small endotherm, with *T*_*o-d*_(H-XIX) = 100 ± 1 K for protiated and *T*_*o-d*_(D-XIX) = 105 ± 1 K for deuterated ice XIX. The larger, second endotherm with *T*_*o-d*_(H-XV) = 131 ± 1 K and *T*_*o-d*_(D-XV) = 126 ± 4 K pertains to protiated and deuterated ice XV domains, respectively. Please note here that the isotope effect is typical for the ice XIX domains, where deuterated domains disorder at higher temperature, but is atypical for the ice XV domains, where deuterated domains disorder at lower temperature. This is a direct consequence of peak overlap between the exotherm and the ice XV endotherm, especially in the deuterated sample. This is explained in more detail later in the manuscript and demonstrated in Fig. S2 in the Supporting Information.

Let us first look at the individual peak areas and hence the kinetically inflicted case as sketched in grey in Fig. 1a. The size of the ice XV endotherm systematically decreases from Δ*H*(H-XV) = 58 ± 9 J mol^−1^ to Δ*H*(D-XV) = 10 ± 7 J mol^−1^ from fully protiated to fully deuterated samples. The entropy difference between ice XV and ice VI, *S*_conf_(XV), can be calculated as *S*_conf_(XV) = Δ*H*(XV)/*T*_*o-d.*_ This then equates to *S*_conf_(H-XV) = 0.44 ± 0.07 J mol^−1^ K^−1^ and *S*_conf_(D-XV) = 0.08 ± 0.05 J mol^−1^ K^−1^. The calorimetrically inferred order (CIO) for protiated and deuterated ice XV in this “single peak approach” amounts to 13% and 2% of the Pauling entropy for ice XV samples cooled at 1.0 GPa, respectively. Especially the latter value represents a significant underestimate due to kinetic infliction that is caused by the overlap of the second endotherm with the exotherm.

Let us now compare this with ice XIX samples prepared by cooling ice VI at 1.8 GPa (Fig. 2b). The first endotherm is very large for three traces (between 5% H_2_O and 100% H_2_O) but small for the fourth trace (red, 0% H_2_O/100% D_2_O). In fact, the first endotherms for the 100% and 95% H_2_O samples are quite similar, whereas the endotherm for the 5% H_2_O/95% D_2_O sample is shifted to slightly higher temperature and is also much broader. This shift reflects the typical H/D-isotope effect noted for many order–disorder transitions of ice polymorphs^17^. The much broader endotherm for 95% deuterated ice XIX implies that disordering takes longer for deuterated ice XIX than for protiated ice XIX. The size of the endotherm for fully deuterated ice XIX amounts to only Δ*H*(D-XIX) = 7 ± 3 J mol^−1^, whereas addition of only 5% H_2_O increases the size of the endotherm to Δ*H*(D-XIX) = 72 ± 5 J mol^−1^. That is, the addition of a small amount of H_2_O increases the enthalpy change and the CIO by approximately a factor of ten. More specifically, in the “single peak approach” *S*_conf_(D-XIX) = 0.07 ± 0.03 J mol^−1^ K^−1^ for fully deuterated ice XIX, but increases to *S*_conf_(D-XIX) = 0.68 ± 0.05 J mol^−1^ K^−1^ after adding 5% H_2_O. That is, the CIO jumps from 2 to 20% from fully deuterated samples to hydrogen-doped deuterated samples. In this context it is important to note that the preparation route for the neutron diffraction experiment reported by Salzmann et al.^11,18^ is the same as the one that leads to 2% CIO here, whereas the one from Gasser et al.^7^ is the one that leads to 20% CIO. In view of this result it is no longer surprising that Salzmann et al. find 0% DIO in the analysis of their neutron data, whereas Gasser et al. find 42% DIO.

### Beyond infliction of kinetics: the thermodynamic picture

In addition to the analysis of three individual, but overlapping peaks, we have also integrated all three peaks together. These results are collected in the column Δ*H*_tot_ in Table 1. The overlap of the three individual peaks can immediately be recognized from the fact that the sum of the three contributions does not equal Δ*H*_tot_ in Table 1. The result in Table 1 that the degree of order is higher for 95% deuterated ice XIX than for protiated ice XIX seems surprising at first look. Also, the strange isotope effect for ice XV mentioned above defies earlier observations in other order–disorder ice pairs. Both observations can be understood quite easily and are a consequence of sluggish kinetics as outlined below. The higher degree of order in H-doped D_2_O ice XIX is a direct consequence from the broader peak (5% H_2_O, green trace in Fig. 2b) than for H_2_O ice XIX (100% and 95% H_2_O, blue and purple traces in Fig. 2b). This broader peak implies slower disordering, and points towards the importance of kinetics for precise determination of the configurational entropy. The “single peak integration approach” merely reflects a kinetic result, whereas in reality we seek for the thermodynamic limit of infinitely slow heating, in which peak overlap is avoided. The issue here is that ice XIX takes a complex two-step path to ultimately turn into disordered ice VI. As shown in our previous neutron, Raman and calorimetry work^7,15,16,19^, ice XIX first transforms to ice XV, via a transiently disordered state ice VI^‡^, and only then transforms to ice VI. The complex calorimetric endotherm-exotherm-endotherm signature in Fig. 2 reflects just that. By simply waiting endlessly below *T*_*o-d*_(XIX) ice XIX remains stable. By waiting just above *T*_*o-d*_ (XIX), but below *T*_*o-d*_ (XV) ice XIX transforms entirely to ice XV. By waiting just above *T*_*o-d*_ (XV) full conversion to ice VI takes place. In such experiments the configurational entropy of ice XIX can be calculated as a sum of two terms: Δ*S*_conf_(ice XIX → VI) = Δ*S*_conf_(XIX → XV) + Δ*S*_conf_(XV → VI). The equilibrium temperature, above which protiated ice XV becomes thermodynamically favoured over ice XIX, was determined to be 103 ± 2 K in our 2018 work on this topic^9^ and is confirmed to be 103 ± 1 K in the present work (column *T*_*o-d*_ (XIX) in Table 1). Protiated ice XV disorders above 131 ± 1 K, in accordance with earlier work^9,20,21^.

In real experiments there is only a finite time for the conversions to take place—in our calorimetry experiments only 3 min are available in the temperature range between 103 and 131 K, where all these transitions occur (at a heating rate of 10 K min^−1^). In these 3 min first the transiently disordered ice VI^‡^ forms and subsequently ice XV forms from ice VI^‡^ and finally ice VI forms from ice XV. At, say 115 K, formation of ice VI^‡^ (causing an endotherm) and formation of ice XV (causing an exotherm) take place simultaneously. At, say 131 K, these processes have not yet finished, but the third process, ice VI formation (causing an endotherm) already kicks in. As a result, three processes take place at the same time. The calorimeter merely records the sum trace (black line in Figs. S1 and S2), whereas it does not record the three fundamental processes at the origin of the sum trace (purple and red endotherms and blue exotherm in Figs. S1 and S2). Depending on the relative kinetics of these processes the sum trace may feature a significant exotherm (as in Fig. S1) or the exotherm may be too small to appear in the sum trace (as in Fig. S2). The sum trace shown in Fig. S1 looks very similar to the 100% H_2_O and 95% H_2_O cases in Fig. 2b, whereas the sum trace in Fig. S2 resembles the 5% H_2_O case. In all these traces the endotherms in the sum trace are in fact somewhat smaller in area than the underlying fundamental endothermic traces. This is owing to the fact that both (fundamental) endotherms overlap with the exotherm—so that parts of the endotherms are missing in the sum trace recorded by the calorimeter. Consequently, the values for CIO and *S*_conf_ calculated in the previous section are merely apparent (kinetic) values, which necessarily underestimate the real (thermodynamic) value. The faster the heating rate the larger the difference between the real thermodynamic and the measured, apparent areas. Furthermore, the overlapping of two fundamental processes results in a shift of the initial temperature for the second endotherm (cf. lower integration limit of dashed red line with lower integration limit of sum trace, marked by arrows in Fig. S1). This shift is more pronounced for the sluggish transitions in deuterated ice than for the faster, narrower transitions in protiated ice. This explains the atypical isotope effect *T*_*o-d*_(D_2_O ice XV) < *T*_*o-d*_(H_2_O ice XV) in Fig. 2a. It is caused simply by overlap of the exotherm with the second endotherm. After peak decomposition and analysis of the second endotherm alone, this isotope effect changes to the typical isotope effect, *T*_*o-d*_(D_2_O ice XV) > *T*_*o-d*_(H_2_O ice XV).

In order to account for the overlapping of peaks and to find a better estimate for the thermodynamic value for *S*_conf_(XIX) it is mandated to integrate not only the individual peaks, but to integrate all three peaks at once, from the onset point of ice XIX disappearance to the end point of ice VI appearance. That is the lower and upper integration boundaries are about 100 K and 145 K in Fig. 2 to determine the overall enthalpy Δ*H*_tot_ taken up for the process ice XIX → ice VI. We refer to this procedure as the “combined peak integration approach”. Please note that the sum of the three individual Δ*H* contributions in Table 1 does not necessarily equal Δ*H*_tot_. This issue is also illustrated in Fig. S2, in which the two individual baselines for the two individual endotherms (dotted grey lines) are different from the baseline of the overall process (full grey line). The two dotted and full grey lines span a triangle, the area of which represents the difference between the standard integration method and the “combined peak integration approach”.

Δ*H*_tot_ can now be used to estimate the thermodynamic limit of CIO and *S*_conf_(XIX). In its exact definition *S*_conf_(ice XIX) = Δ*H*(XIX → XV)/*T*_*o-d*_(XIX) + Δ*H*(XV → VI)/*T*_*o-d*_(XV). Unfortunately our thermodynamic approach here does not allow us to access Δ*H*(XIX → XV) and Δ*H*(XV → VI) individually, but only the sum Δ*H*_tot_ = Δ*H*(XIX → XV) + Δ*H*(XV → VI). To approximate the exact term above we simplify it to *S*_conf_(ice XIX) = Δ*H*_tot_/*T*_*o-d*_(XIX), where *T*_*o-d*_(XIX) is used instead of *T*_*o-d*_(XV) for the second term. Since *T*_*o-d*_(XIX) < *T*_*o-d*_(XV) this means that our estimate for the thermodynamic limit of *S*_conf_ is actually an overestimation or upper limit. Table 1 helps to estimate by how much we overestimate the thermodynamic limit. For protiated ice XIX the onset temperatures are 103 and 128 K, and the enthalpies are 50, − 6 and 44 J mol^−1^, respectively. For this case *S*_conf_(ice XIX) = 44/103 + 44/128 = 0.77 J mol^−1^ K^−1^. Our estimate for this case is *S*_conf_(ice XIX) = 87/103 = 0.84 J mol^−1^ K^−1^. Based on this thermodynamic estimate the (upper limit) CIO is 25 ± 3% for fully protiated ice XIX and 5 ± 2% for fully deuterated ice XIX from the data in Table 1. This compares with 12% CIO and 2% CIO from the kinetic, single-peak integration approach. For 5% protiated D_2_O ice XIX the CIO is 20% and the DIO 42%.

In case of ice XV the thermodynamic approach is done by using the Δ*H*_tot_ and *T*_*o-d*_(XV) columns in the ice XV section of Table 1. This results in 9 ± 4% CIO for ice XV, i.e., less than half of the CIO in ice XIX: This higher degree of hydrogen order is the basis for ice XIX to be the thermodynamically stable low-temperature phase in the intermediate pressure range. This claim is elaborated in more detail in the subsequent sections. For deuterated ice XV the CIO is 6 ± 2% (instead of 2% in the kinetic approach), i.e., it drops considerably less than in ice XIX. This leads to the question of which mechanism is responsible for the large kinetic inhibition in deuterated ice XIX, but much smaller inhibition in deuterated ice XV. In our recent kinetic analysis work^12^ we have suggested quantum tunnelling to be a possible reason: We suggest quantum tunnelling does not affect the ice XV/VI transition very much at 130 K, but does affect the ice XIX/VI^‡^ transition at 100 K. Here quantum tunnelling does increase the mobility of H-atoms significantly, but not so much the mobility of the twice as heavy D-atoms.

### Using the Clapeyron equation to infer the initial slope of the XIX/VI phase boundary

**Figure 3:**
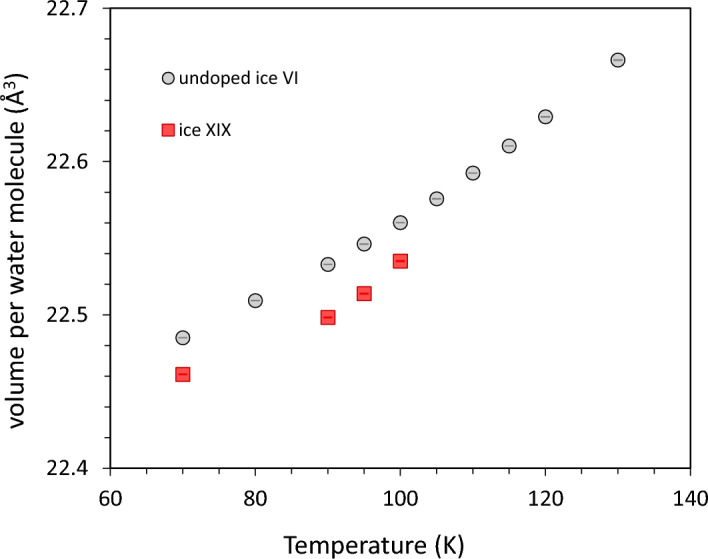
Volume per water molecule for ice XIX and ice VI as deduced from HRPD measurements, where the volume of the unit cell was divided by 10 molecules/unit cell for ice VI; and divided by 20 molecules/unit cell for ice XIX.

**Figure 4:**
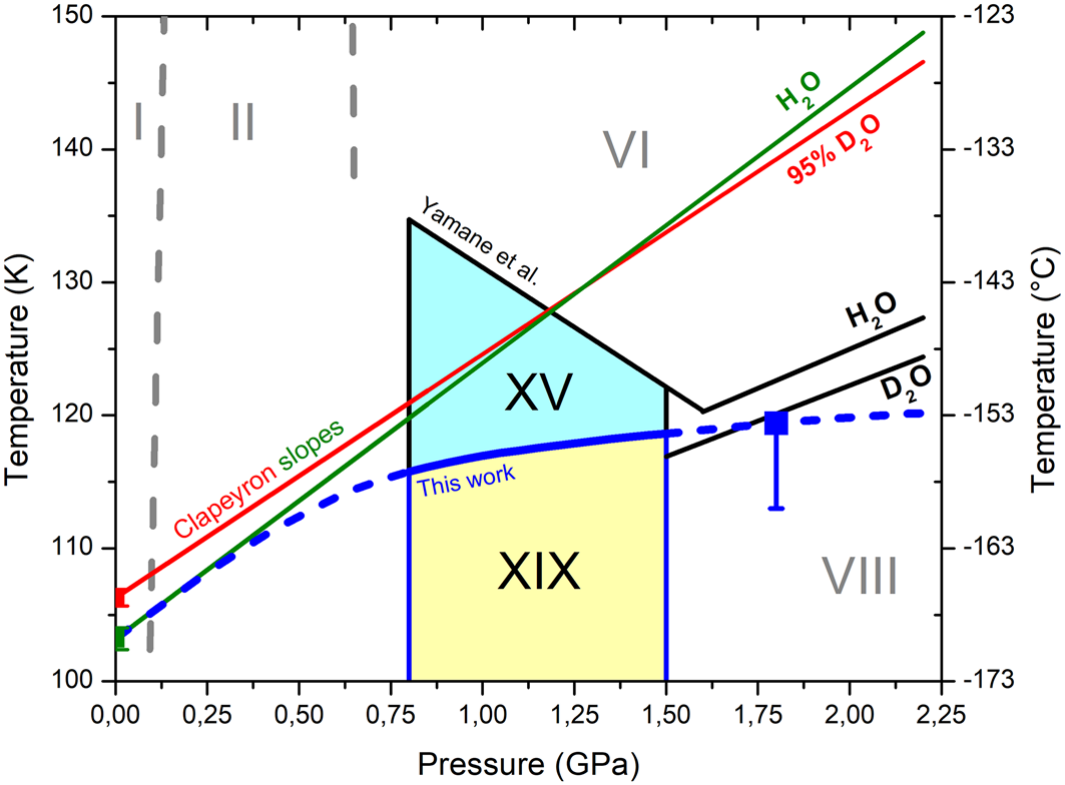
Estimate for the location of the H_2_O ice XIX phase boundary (blue dashed and full line) and the thermodynamic stability range of ice XIX (yellow area) and ice XV (light blue area). This estimate is based on (i) the calorimetric order–disorder temperature at 0.0 GPa (green square), (ii) the Clapeyron slope at ambient pressure (green line) and (iii) the high-pressure experiment at 1.8 GPa outlined in Fig. 5 (blue square). The ice XV and ice XIX phase boundaries estimated by Yamane et al. in ref.^8^ from in situ dielectric experiments are shown as black lines. The calorimetric order–disorder temperature and Clapeyron slope for D_2_O ice XIX are shown as red square and red line, respectively, where the intersection near 1.2 GPa explains the reversal of the isotope effect at 1.6 GPa.

The *T*_*o-d*_ values listed in Table 1 are strictly valid only for ambient pressure conditions (1 bar, 0.0 GPa). It is now of interest to evaluate how the phase boundary between ice XIX and ice VI, i.e., *T*_*o-d*_(XIX), changes with pressure. To determine the slope of the phase boundary d*T*/d*p* in the thermodynamic limit according to the Clapeyron equation both the volume difference and the entropy difference need to be known. The thermodynamic limit for the entropy difference has been described in the previous section. The specific volume difference is inferred from our previous neutron diffraction experiments at subambient pressure^1^. Even though the Clapeyron slope is strictly valid only for 0.0 GPa, it can still be extrapolated to higher pressure. Typically, the slope is rather pressure insensitive for order–disorder transitions because there is usually no volume change associated with the transition. Neutron diffraction experiments, however, have shown that there are small differences in the densities of the ice VI, XV, XIX trio. Figure 3 shows the volume per water molecule in the unit cells of ice XIX and ice VI based on our neutron powder diffraction as a function of temperature. Between 70 and 100 K ice XIX is denser than ice VI. The difference is extremely small (~ 0.12%) but highly statistically significant considering the precision of the lattice parameters refined from the high-resolution powder diffraction data; the mean absolute difference in molecular volume over the range 70–100 K is 0.029 ± 0.005 Å^3^. Based on the Clapeyron equation this implies that the onset temperature for ice XIX disordering increases with pressure, i.e., d*T*/d*p* is necessarily positive for the ice XIX/VI phase boundary. By contrast ice XV is slightly less dense than ice VI^22^, and so d*T*/d*p* is negative for the ice XV/VI phase boundary. A positively sloped ice XIX and a negatively sloped ice XV phase boundary were also determined by Yamane et al.^8^ at > 0.8 GPa (see black lines marked H_2_O in Fig. 4).

Let us now quantify the Clapeyron slope for ice VI/XIX by combining calorimetric entropy data with neutron volume data using the equation d*T*/d*p* = (*V*_m,VI_–*V*_m,XIX_)/(*S*_m,VI_–*S*_m,XIX_). The molar volume difference between ices VI and XIX per water molecule scatters between + 0.024 and + 0.035 Å^3^ in the range between 70 and 100 K in Fig. 3. That is, *V*_m,VI_–*V*_m,XIX_ = 17 ± 3·10^−9^ m^3^ mol^−1^. We here assume that this difference is independent of isotopologue, i.e., the same for H_2_O and D_2_O. The molar entropy difference between ices VI and XIX (also at 0.0 GPa) from Table 1 amounts to *S*_m,VI_–*S*_m,XIX_ = 0.843 J mol^−1^ K^−1^, when taking the (upper) thermodynamic limit from the “combined peak integration approach”. Substituting these entropy and volume differences into the Clapeyron equation yields d*T*/d*p* =  + 21 ± 5 K/GPa for protiated ice XIX and d*T*/d*p* =  + 18 ± 4 K/GPa for deuterated ice XIX (with 5% H). That is, the phase-boundary for deuterated ice XIX is flatter than the phase-boundary for protiated ice XIX, but starts at higher temperature (108 ± 2 K vs 103 ± 1 K, see Table 1). This means that the two phase boundaries cross at some pressure as shown in Fig. 4, which we determine to be roughly at 1.2 GPa from the data in this work. However, the error-bar on this value is quite high since the difference between the slopes is smaller than a single standard deviation. Yet, our result suggests reversal of the isotope effect at high-pressure conditions. This agrees very well with the measurements by Yamane et al., who determined the order–disorder temperatures from high-pressure dielectric measurements and who (maybe surprisingly) found that the disordering temperature is lower for D_2_O ice XIX at 1.6 GPa than for H_2_O ice XIX (cf. black lines labelled H_2_O and D_2_O in Fig. 4).

### The ice XIX phase boundary at 1.8 GPa from a high-pressure experiment

**Figure 5:**
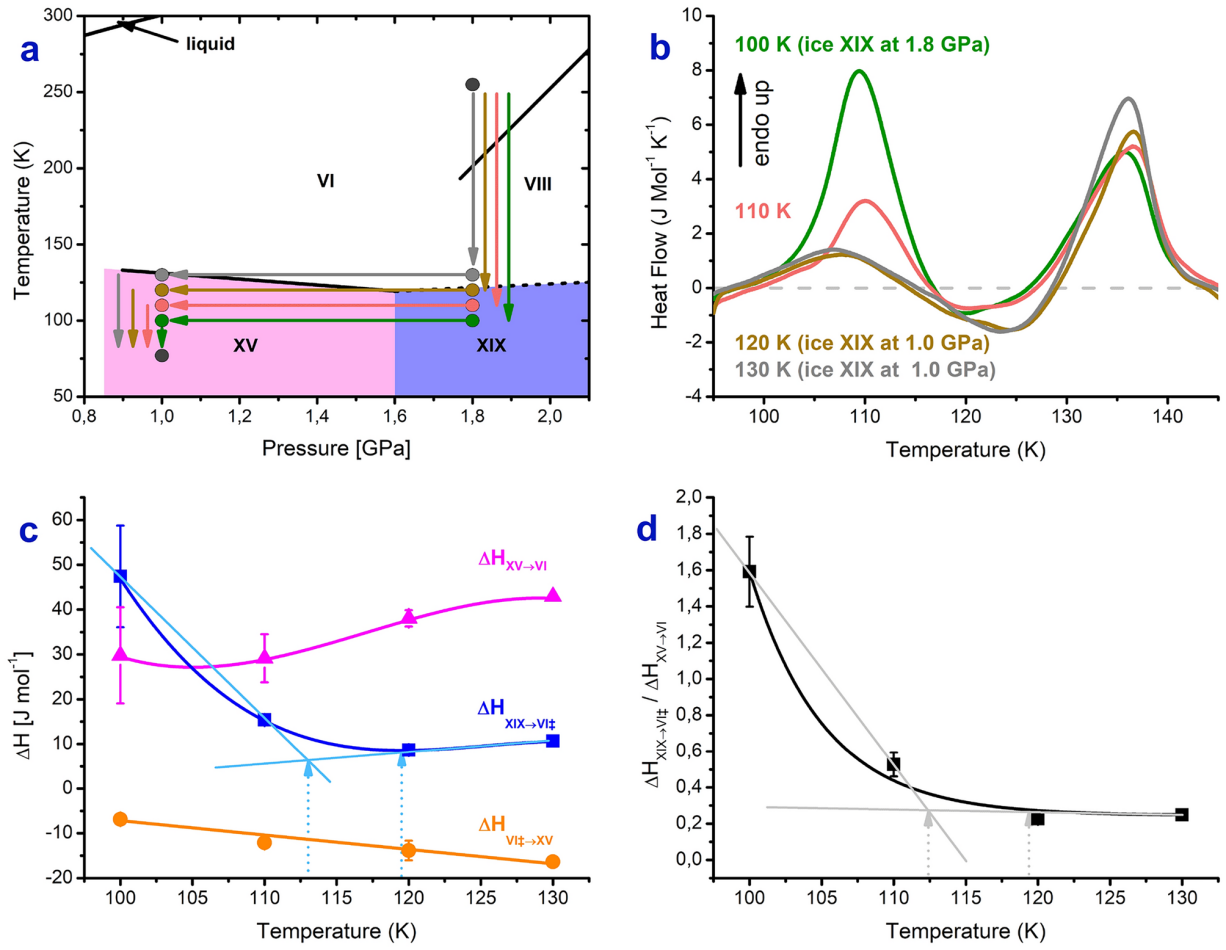
Bracketing experiment to obtain the location of the ice XIX phase boundary at 1.8 GPa using a 95% H_2_O and 5% D_2_O sample. (**a**) Schematic representation of experimental paths starting from ice VI at 1.8 GPa and 255 K. Cooling rates are 3 K min^−1^ at 1.8 GPa and ≈60 ± 10 K min^−1^ at 1.0 GPa. The rate of decompression is − 0.1 GPa min^−1^. (**b**) Calorimetry traces recorded upon heating recovered samples at 10 K min^−1^ at ambient pressure. The dashed grey line represents the baseline. Endothermic peaks are pointing upwards. (**c**) Transformation enthalpies for the ice XIX endotherm (blue), ice VI^‡^ exotherm (orange) and ice XV endotherm (purple). (**d**) Ratio of the areas for the ice XIX and ice XV endotherms. Lines in (**b**) and (**c**) are guides to the eye. In (**c**) and (**d**) the light blue and grey lines are used to estimate the upper and lower limit for *T*_*o-d*_(XIX, 1.8 GPa).These estimates are marked with light blue and grey dashed arrows. We regard the upper limit as the most likely value because this leads to a more natural, smooth curve.

Figure 5a shows the design of a bracketing experiment to estimate the order–disorder temperature for ice XIX at 1.8 GPa, *T*_*o-d*_(1.8 GPa). Ice VI is cooled at 1.8 GPa to different temperatures, namely 130 K, 120 K, 110 K and 100 K. The sample is then decompressed isothermally at these temperatures to 1.0 GPa, where it is quenched to 77 K and recovered. If the temperature is above *T*_*o-d*_(1.8 GPa) ice VI remains at 1.8 GPa, which in turn transforms to ice XV when quenching at 1.0 GPa. If on the other hand the temperature is below *T*_*o-d*_(1.8 GPa) ice XIX is produced upon cooling at 1.8 GPa. Ice XIX then remains unaffected upon decompression and quenching at 1.0 GPa. The calorimetry scans for the quench-recovered samples shown in Fig. 5b provide the answer about *T*_*o-d*_(1.8 GPa): for temperatures of 130 K and 120 K barely any ice XIX forms. The first endotherm amounts to about 10 J mol^−1^, which is typical of ice XV samples prepared at 1.0 GPa (cf. Fig. 2a and Table 1). This implies that *T*_*o-d*_(1.8 GPa) < 120 K. By contrast, the first endotherm is clearly larger for decompression temperatures of 110 K and 100 K, respectively (see Fig. 5b). As seen in Fig. 5c (blue line) the first endotherm increases slightly to 15 J mol^−1^ at 110 K and then massively to 50 J mol^−1^ at 100 K. At 100 K approximately the values are reached that are given in Table 1 for protiated ice XIX. In other words, after cooling to 100 K at 1.8 GPa ice XIX has fully developed. After cooling to 110 K at 1.8 GPa some ice XIX has already developed, which signifies *T*_*o-d*_(1.8 GPa) > 110 K. This calorimetric result is also confirmed from the X-ray powder diffractograms shown in Fig. S5. As demonstrated in our earlier work^9^ the Bragg peak near *d*-spacings of 0.265 nm shifts to slightly lower distances in ice XIX compared to ice XV. Such a shift is seen at 110 K, whereas at 120 K and 130 K the Bragg peak is at its characteristic position for ice XV. Based on the blue curve in Fig. 5c and the curve defining the ratio Δ*H*(XIX)/Δ*H*(XV) in Fig. 5d the upper and lower limits for *T*_*o-d*_(1.8 GPa) are 119 K and 113 K, respectively, using the tangent intersection method (see Fig. 5c, d, dotted arrows). We regard 119 K as the best estimate because it avoids sharp kinks in the ice XIX enthalpy and ice XIX/XV enthalpy ratio curves in Fig. 5c and d. An even better bracketing would require a narrower spacing of temperatures between 110 and 120 K. *T*_*o-d*_(1.8 GPa) is about 13 ± 3 K higher than the corresponding value at ambient pressure, *T*_*o-d*_(0.0 GPa) = 103 ± 1 K. Based on the two experimentally determined data points for *T*_*o-d*_(XIX) the average slope of the ice XIX/VI phase boundary between 0.0 and 1.8 GPa is d*T*/d*p* = 7 ± 2 K GPa^−1^. This average slope over the whole pressure range is significantly smaller than the initial slope at ambient pressure d*T*/d*p* = + 21 ± 5 K GPa^−1^determined using the Clapeyron approach above. This implies that the phase-boundary is in reality not a straight line (as suggested in Fig. 4), but flattens off at high-pressure conditions. Our slopes here compare with a slope of about 12 K GPa^−1^ measured by Yamane et al. above 1.5 GPa (cf. black line in Fig. 4). While the slopes determined by them using dielectric measurements are similar to ours, our measurements suggest that the slope above 1.5 GPa is < 7 K GPa^−1^. The slope at high-pressure conditions has to be smaller than the average of 7 K GPa^−1^ if the slope at low-pressure conditions is + 21 ± 5 K GPa^−1^—see the dashed blue line in Fig. 4 that initially follows the Clapeyron slope (green line). This is reflected in Fig. 4, where the high-pressure portion of our phase boundary (dashed blue line) is flatter than the phase boundary by Yamane et al.

The ice XIX/VI phase boundary is especially relevant in the pressure range between 0.8 and 1.5 GPa because ice XIX is the thermodynamically stable phase below *T*_*o-d*_(XIX/VI) there. For this reason the blue line is depicted as a full line in this pressure range. Our results suggest that the phase-boundary is not exactly parallel to the temperature axis as depicted in Fig. 1 in ref.^9^. It is also not located at 103 K as surmised in our earlier work^1^. Based on the data obtained in the present work the phase boundary in the phase diagram should increase weakly with pressure, from 0.8 GPa/115 ± 2 K to 1.5 GPa/118 ± 2 K, thereby defining the area of thermodynamic stability in the phase diagram (yellow area in Fig. 4). Above this line ice XV is thermodynamically stable (blue area in Fig. 4).

## Discussion and conclusions

In this work we present a study aimed at determining the calorimetrically inferred degree of hydrogen ordering (CIO) in ice XV and ice XIX. This is especially challenging because ice XIX disorders in a way that has not been observed for any other ice polymorph in the past. The complexity arises from the fact that ice XIX starts to disorder at 103 ± 1 K, but then simultaneously its hydrogen-ordered sibling, ice XV, forms from the disordered state, before ice XV ultimately disorders to produce ice VI. All steps in this sequence take place at a specific rate, so that kinetic aspects such as the heating rate in the calorimetry instrument play a decisive role as evidenced in Fig. 1. This results in significant overlap of the associated thermal signatures. Overlap poses a challenge in assessing the degree of order, where we here compare two types of analysis methods: the single peak integration and the combined peak integration approaches. The former does not account for peak overlap at all and so merely reflects a kinetically inflicted result that inevitably changes with choice of heating rate. The latter is independent of heating rate and avoids overlaps because only the difference between the initial state, ice XIX, and the final state, ice VI, is accounted for. Thus, the combined peak integration approach estimates the thermodynamic limit (of infinitely slow heating) for the enthalpy difference between ice XIX and ice VI, where infinitely slow heating experiments cannot be realized in practice because of the signal being proportional to the heating rate. In case of the ice XIX/XV/VI transition sequence this thermodynamic approach as defined by us results in an upper limit of the CIO.

This approach shows that the CIO for ice XIX prepared at 1.8 GPa is about one quarter of the full Pauling entropy, but double the value for ice XV prepared at 1.0 GPa. This higher degree of ordering favours ice XIX over ice XV and ice VI at low temperatures, so that ice XIX is the thermodynamically stable phase in the intermediate pressure range (see Fig. 4). The location of the phase boundary (blue line in Fig. 4) is estimated in our work from (i) the onset temperature of disordering in the calorimetric experiment of 103 ± 1 K at 0.0 GPa, (ii) the Clapeyron slope of 21 ± 5 K GPa^−1^ at 0.0 GPa as calculated from the CIO and the neutron-diffraction based volume difference and (iii) the ordering temperature of ice XIX of 116 ± 3 K measured at 1.8 GPa using the bracketing experiment shown in Fig. 5. The location of the phase boundary is quite similar in our work to the one determined in the dielectric study by Yamane et al.^8^. Our initial Clapeyron slope at 0.0 GPa is twice as high as the one by Yamane et al. above 1.5 GPa, but our high-pressure slope drops below the values reported by Yamane et al.

Furthermore, we systematically study the influence of H/D isotope exchange on the transition sequence (summarized in Table 1). In general, deuteration slows down both the ordering and disordering kinetics of the H/D sublattice. However, this effect is much more massive for ice XIX than for ice XV. In the case of ice XIX, full deuteration slows down the kinetics so much that the CIO in fully deuterated ice XIX is only 5% compared to 25% in fully protiated ice XIX. In the case of ice XV prepared at 1.0 GPa, by contrast, only a small drop from 9 to 6% is observed. This large kinetic isotope effect on the degree of ordering in ice XIX also affects the Clapeyron slope of the phase boundary, where deuteration reduces the slope, but deuteration elevates the order–disorder temperature *T*_*o-d*_(XIX) at ambient pressure. As a consequence, the isotope effect apparently reverses from *T*_*o-d*_(H-XIX) < *T*_*o-d*_(D-XIX) at ambient pressure to *T*_*o-d*_(D-XIX) < *T*_*o-d*_(H-XIX) above 1.5 GPa. This explains the surprising observation made by Yamane et al. in their in situ dielectric work^8^. Future high-precision measurements of the volume difference between ice VI and ice XIX at high pressure and direct observation of the ice XIX/VI phase-transformation at high-pressure conditions will be useful to exactly measure the phase boundary and to reduce the error-bars encountered in the present work. Such measurements could, e.g., be done using neutron diffraction at the MITO system on the PLANET beamline at J-PARC^23–25^. Before such measurements are done we regard the stability fields of ice XIX and ice XV shown in Fig. 4 to be the best data available. Yet, they rely on calculated Clapeyron slopes, a single bracketing experiment at 1.8 GPa and hence might be refined in future works once more data become available.

By contrast to ice XIX the degree of deuterium order can be enhanced in ice XV by heating and recooling at ambient pressure^15,21,22,26^. This fact has immediately allowed for the neutron structure elucidation for ice XV, whereas for ice XIX ways around the kinetic inhibition for deuterated ice XIX had to be found first. The key ingredient there is introduction of fast H-atom defects, which allows to obtain 28% of the Pauling entropy as opposed to almost zero order in fully deuterated samples. In the in situ neutron diffraction experiments reported by Salzmann et al. at 1.6 GPa full deuteration was used^11^, and so it is unsurprising that they have determined a disordered crystal structure for what they incorrectly call “ice XIX”. The issue that the Salzmann-ice XIX is not really the ice XIX described by us has already been brought up by Hansen^27^ and later-on substantiated by Komatsu^12^. The higher degree of order in deuterated samples containing H-defects is comparable with the neutron diffraction results by Yamane et al.^8^ and by ourselves^7^, showing a neutron-diffraction inferred ordering (DIO) of up to 42%. This role of hydrogen atoms as dopant to speed up deuterium sublattice dynamics massively is fascinating and has not been noted previously for any other ice polymorph. This certainly prompts for more work on the question.

### Supplementary Information

Supplementary Information.

## Data Availability

Data is provided within the manuscript or supplementary information files. Raw data can be obtained from the corresponding author, Thomas Loerting, upon reasonable request.
