# Supplementary material for: Configurational entropy of ice XIX and its isotope effect
Source: Sci Rep. 2024 May 7;14:10517. doi: 10.1038/s41598-024-61250-9 (PMC11076531; doi:10.1038/s41598-024-61250-9)
Supplement: Supplementary file 1 — Supplementary Information. [file 41598_2024_61250_MOESM1_ESM.docx]

# **SUPPORTING INFORMATION**

**Configurational Entropy of Ice XIX and its Isotope Effect**

Tobias M. Gasser^1^, Alexander V. Thoeny^1^, A. Dominic Fortes^2^, Thomas Loerting^1,^*

^1^*Institute of Physical Chemistry, University of Innsbruck, 6020 Innsbruck, Austria*

^2^*ISIS Neutron and Muon Facility, Rutherford Appleton Laboratory, Harwell Science and Innovation Campus, Chilton, Oxfordshire, OX11 0QX, UK*

*e-mail: thomas.loerting@uibk.ac.at*

In the Supporting Information we show why integration of single peaks (called “single peak approach” in the main manuscript) is problematic and how overlapping of the exotherm with both endotherms shifts the peak onset temperatures and changes the peak areas(Figs. S1 and S2). Furthermore we report X-ray powder diffractograms for all samples that were used to measure the calorigrams in the main manuscript (Figs.S3-S5).

Fig.S1 shows the three individual contributions to the heat flow signal, where both endotherms are well separated, but the exotherm overlaps with both endotherms. This is representative of the situation that we find for pure water samples in Fig. 1 of the main manuscript. Specifically we have assumed two endotherms of +65 and +50 J mol^-1^ (pink and dashed red line) and one exotherm of -50 J mol^-1^ (dashed blue line). These three features overlap significantly, especially since the exothermic formation of ice XV is a very slow process. The black dotted sum curve in Fig.S1 is very similar to what we observe in Fig.1b. Integration of only the first endotherm results in only +55 J mol^-1^, i.e., underestimates the correct value by about 15%. The exotherm in the sum curve integrates to -30 J mol^-1^, which underestimates the real value by 40%, and the second endotherm in the sum curve alone integrates to 40 J mol^-1^, an underestimation of 10%. This is because of the annihilation through simultaneous heat generation and heat uptake in the experiment. Futhermore, the onset temperature for the second endotherm is significantly shifted between the black sum curve and the individual red-dashed contribution.

The situation encountered for deuterated ice XIX (95%H/5%D) is schematically represented in Fig.S2. In comparison to the case of H_2_O ice XIX in Fig.S1 all individual peak contributions are here much broader because of the sluggish movement of D atoms in comparison to H atoms. This leads to an additional overlapping between the two endotherms – which means that at 120 K all three processes generating heat take place simultaneously: disordering of ice XIX, formation of ice XV and disordering of ice XV. This leads to the situation that the exotherm is not even detected in the black sum curve under these circumstances, even though an exotherm of -30 J mol^-1^ is in fact present (blue dashed curve). This then leads to problems with determination of integration boundaries and definition of the baseline. Peak integration for the two endotherms is typically done using tilted lines (grey dashed line), instead of the flat baseline (full grey line). Summing up the two endotherms results then in a total enthalpy of +55 + 14 = 69 J mol^-1^, whereas the real total enthalpy would be the sum of the three individual contributions, ΔH_tot_ = 90 – 30 + 30 = 90 J mol^-1^. The choice of incorrect baselines in highly overlapping peaks is the fundamental issue that causes the incorrectly derived total peak area. For this reason, the real thermodynamic limit can only be accessed by integrating all peaks in a single step, using a single basis – this is called the “combined integration approach” and superior to the “single peak approach”.


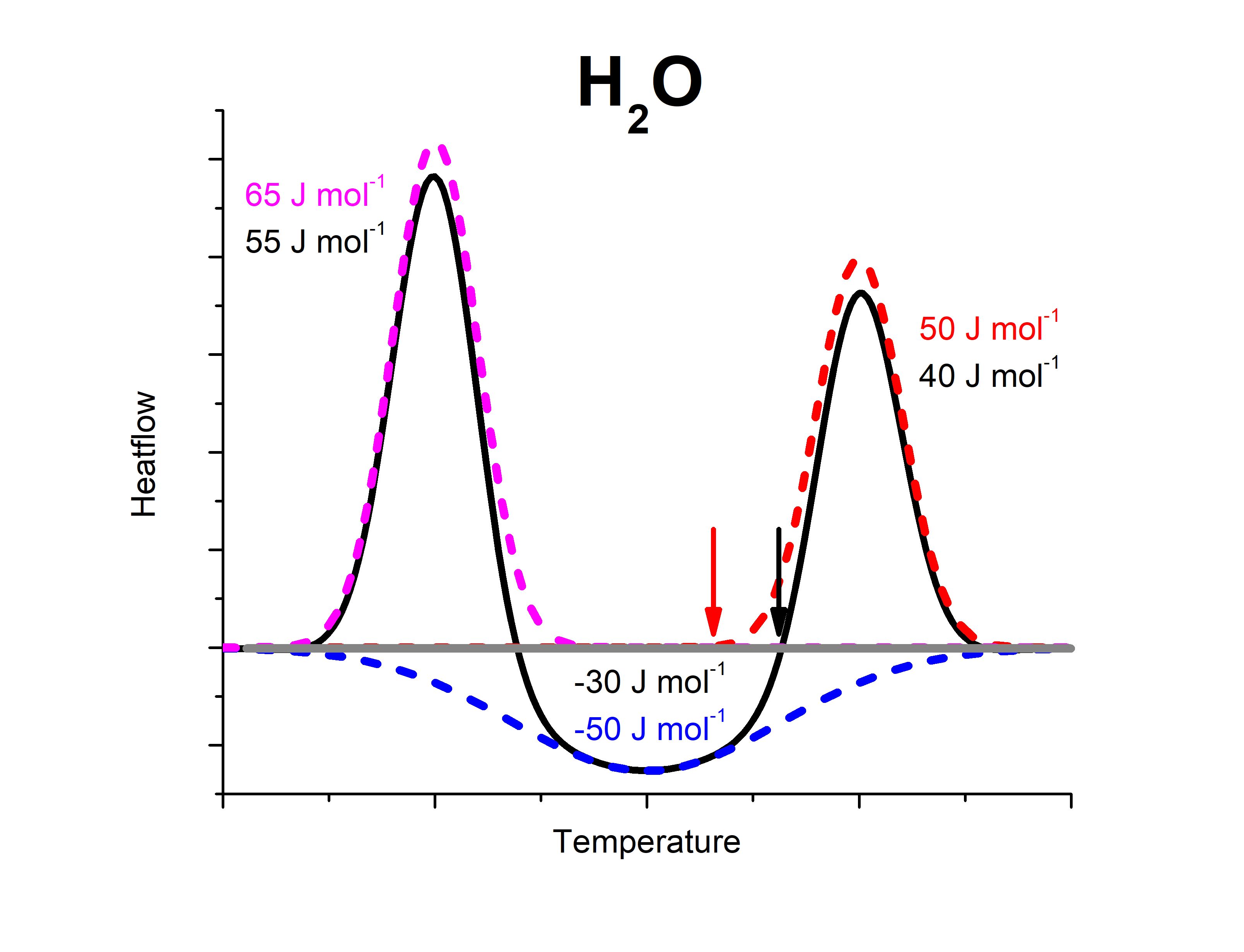


**Figure S1**: Constructed sum thermogram for the case of an H_2_O ice XIX sample (black line) calculated from three individual, but overlapping events. The pink dashed endotherm represents ice XIX disordering, the red dashed endotherm represents ice XV disordering and the blue dashed exotherm represent ice XV formation from transient ice VI^‡^. The grey line represents the baseline of the scan. Arrows are marking the lower integration limits of the second endotherm of the deconvoluted traces (red) and the sum trace (black).


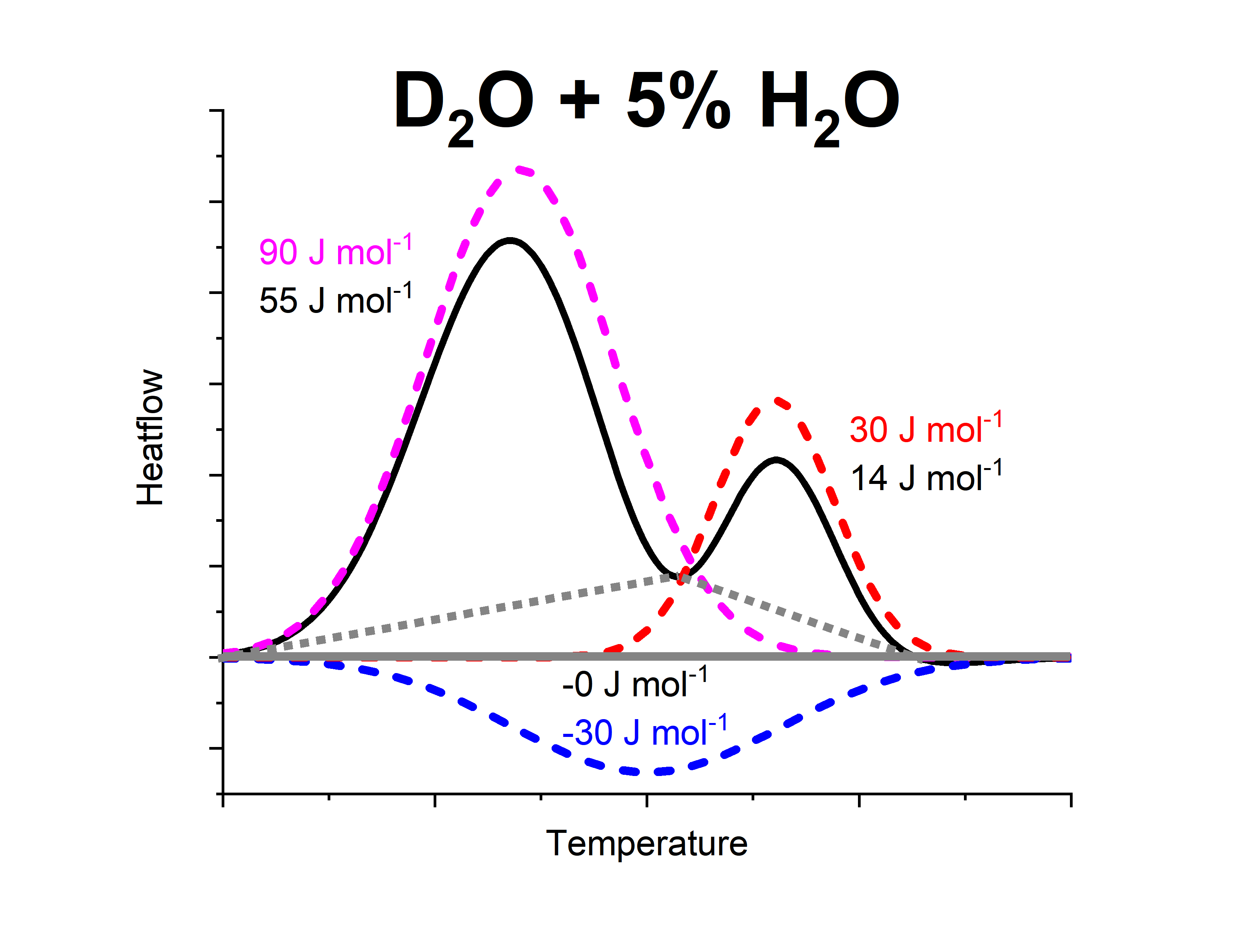


**Figure S2**: Constructed sum thermogram for the case of a D_2_O ice XIX sample (black line) calculated from three individual, but overlapping events. The pink dashed endotherm represents ice XIX disordering, the red dashed endotherm represents ice XV disordering and the blue dashed exotherm represent ice XV formation from transient ice VI^‡^. Compared to Fig. S1 all peaks are broader because rearrangements of D atoms are more sluggish than rearrangements of H atoms. The full grey line represents the baseline of the measurement. The grey dotted lines represent the incorrect baselines typically chosen for integration of the two endotherms. The choice of such incorrect baselines then falsifies the total peak area, calculated as sum of the two endotherm areas.


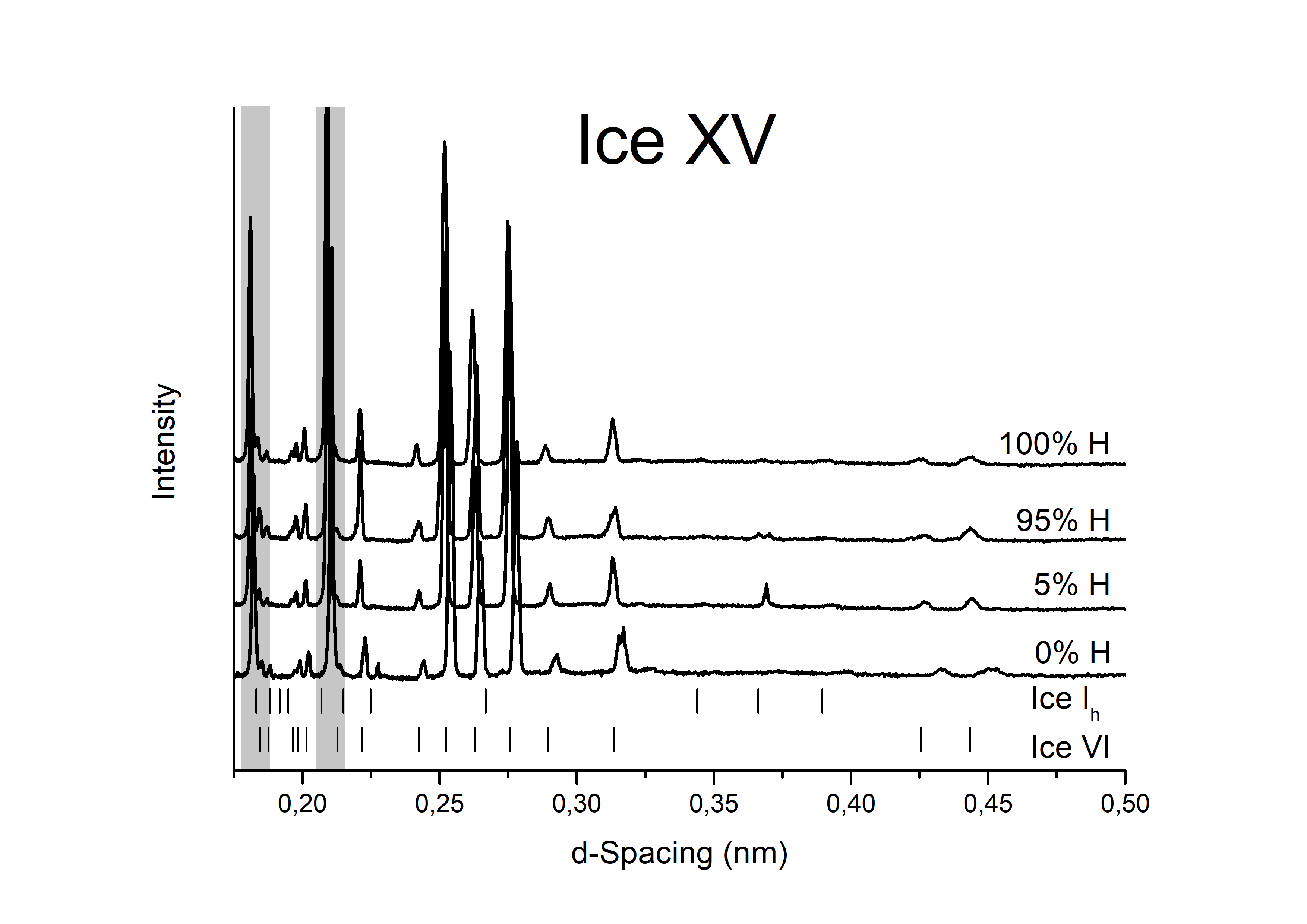


**Figure S3**: X-ray powder diffractograms of the sample batch used for obtaining the data in Fig. 1a of the main manuscript. The pattern was measured using a Bruker D8 powder diffractometer. Grey shaded areas mark the region of the Cu sample holder peaks. Ice I_h_ ^21^ and ice VI ^22^ sample reflex positions are indicated as tick marks.


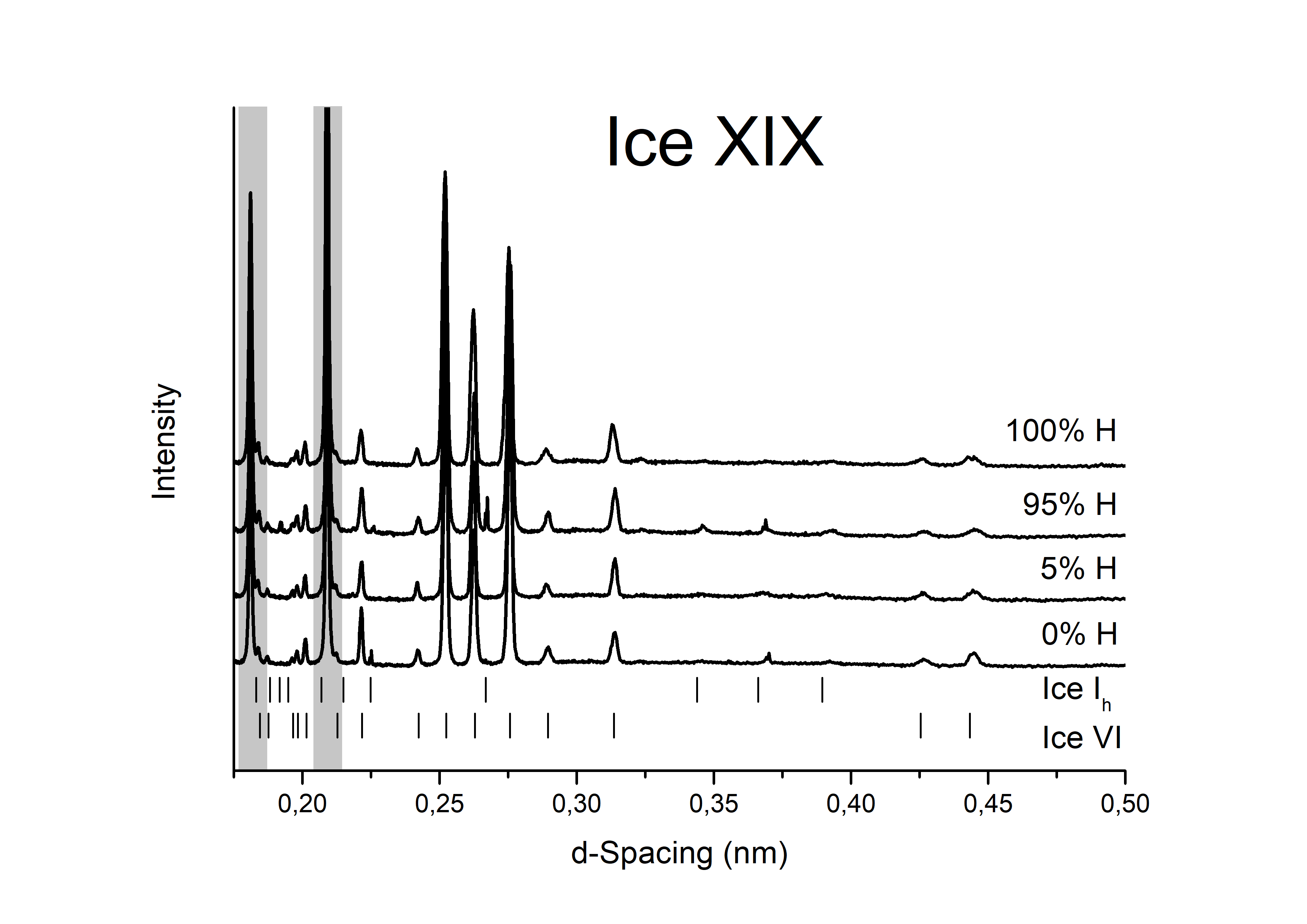


**Figure S4**: X-ray powder diffractograms of the sample batch used for obtaining the data in Fig. 1b of the main manuscript. The pattern was measured using a Bruker D8 powder diffractometer. Grey shaded areas mark the region of the Cu sample holder peaks. Ice I_h_ ^21^ and ice VI ^22^ sample reflex positions are indicated as tick marks.


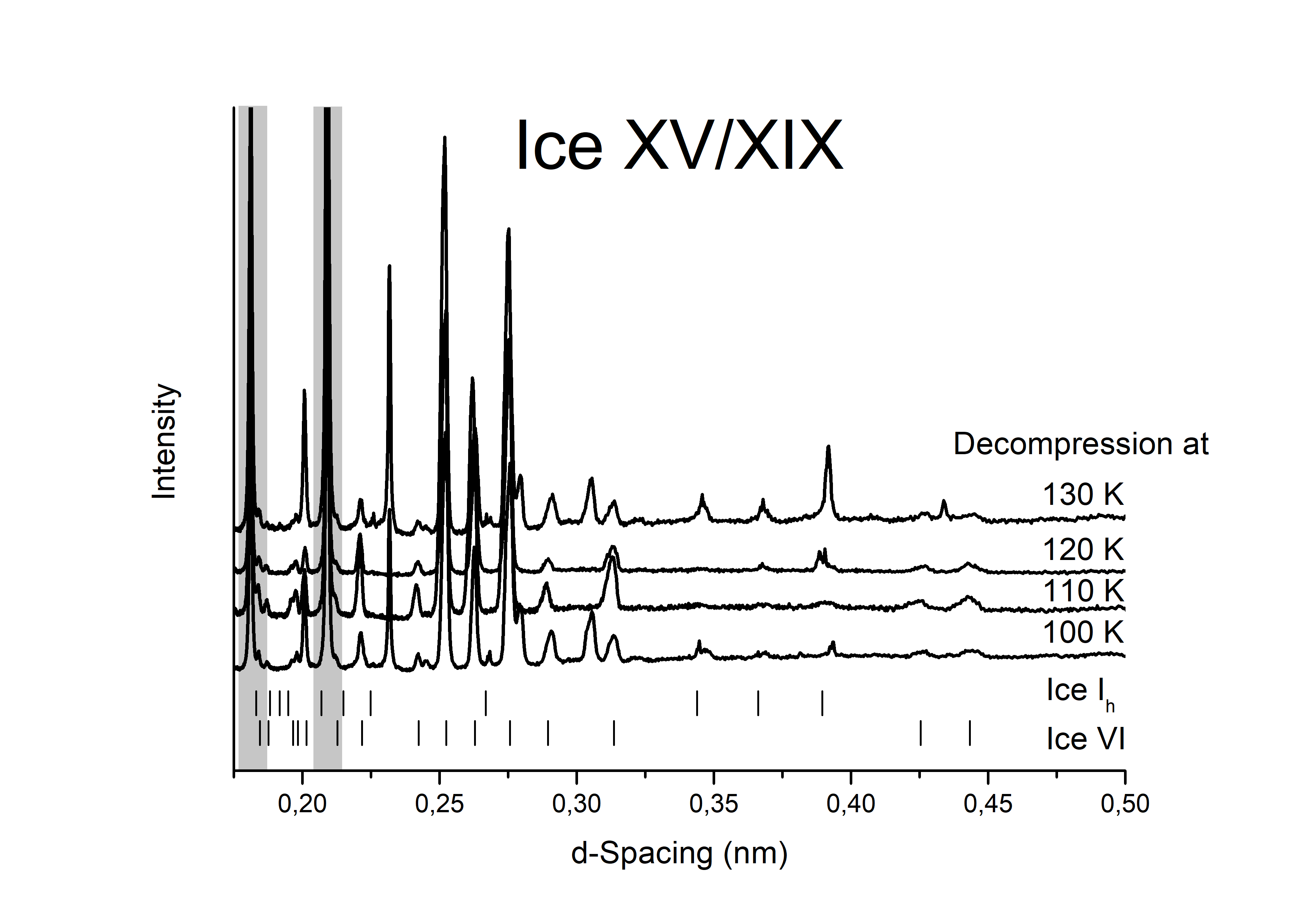


**Figure S5**: X-ray powder diffractograms of the sample batch used for obtaining the data in Fig. 4 of the main manuscript. The pattern was measured using a Bruker D8 powder diffractometer. Grey shaded areas mark the region of the Cu sample holder peaks. Ice I_h_ ^21^ and ice VI ^22^ sample reflex positions are indicated as tick marks.
